# Sol–gel synthesis and solar photocatalytic activity of Ca-alloyed ZnO nanoparticles elaborated using different precursors

**DOI:** 10.1039/c9ra10131d

**Published:** 2020-07-06

**Authors:** A. Rosset, K. Djessas, V. Goetz, S. Grillo, G. Plantard

**Affiliations:** Laboratoire Procédés Matériaux et Energie Solaire, PROMES-CNRS, Rambla de la Thermodynamique, Technosud 66100 Perpignan Cedex France aurelie.rosset0868@gmail.com djessas@univ-perp.fr; Université de Perpignan Via Domitia 52 avenue Paul Alduy 66860 Perpignan Cedex 9 France

## Abstract

Ca-alloyed ZnO nanoparticles elaborated using different calcium precursors (CaSO_4_, CaCl_2_, Ca(NO_3_)_2_ and CaCO_3_) at different [Ca]/[Zn] ratios (0, 1, 5, 10, 15 and 20%) have been prepared by a sol–gel method followed by supercritical drying and annealing at 300 °C. The synthesized samples have been characterized by a number of techniques including Scanning Electron Microscopy (SEM), Transmission Electron Microscopy (TEM), Energy Dispersive X-ray Spectroscopy (EDS), X-ray Diffraction (XRD), Raman Spectroscopy and Electron Paramagnetic Resonance (EPR). SEM and TEM images reveal that the nanoparticles have a quasi-spherical shape with a grain size between 20 and 40 nm. The EDS results on chemical elementary compositions show that the Ca-alloyed ZnO with a CaCO_3_ precursor and [Ca]/[Zn] ratios of 5 and 10% are quasi-stoichiometric. The XRD results indicate that all the elaborated nanoparticles have a hexagonal wurtzite structure. Using Raman Spectroscopy a supplementary vibrational mode is detected in the case of CaSO_4_, CaCO_3_ and Ca(NO_3_)_2_ precursors. The intrinsic defect centers and defect number have been studied using EPR. Two intrinsic defects with different *g* factors are identified by EPR for which the spectral intensities change with calcium precursors. Furthermore, EPR reveals a correlation between the defect number and photocatalytic efficiency. The photocatalytic efficiency of the nanoparticles elaborated with different precursors and compositions has been studied for the solar photocatalytic degradation of pyrimethanil, using a solar simulator. The results show that the nanoparticles of Ca-alloyed ZnO elaborated with a CaCO_3_ precursor give promising results and enhance the photocatalytic efficiency in the solar field.

## Abstract 2

Schematic representation of core–shell intrinsic defects in Zn_1−*x*_Ca_*x*_O nanoparticles observed by EPR spectroscopy.

## Graphical abstract

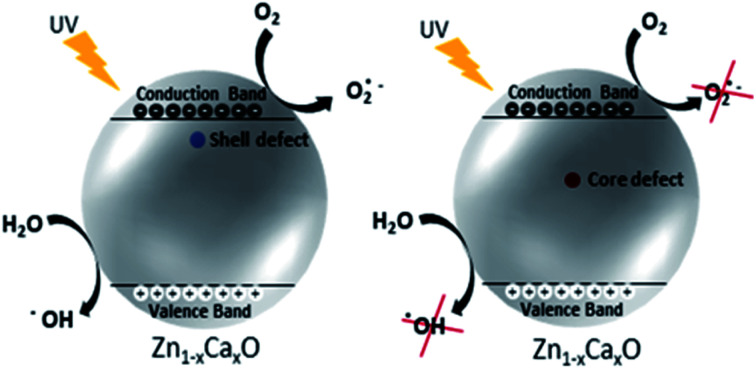

## 1. Introduction

Heterogeneous solar photocatalysis belongs to the family of Advanced Oxidation Processes (AOPs), which are respectful of the environment. This process allows mineralization of a great number of organic pollutants in water thanks to the production of hydroxyl radicals. During the last decade it has attracted considerable attention.^1^ Photocatalytic activity occurs when the semi-conductor absorbs a photon with energy equal to or greater than the material's band gap, leading to the formation of electron and hole pairs. These pairs can subsequently migrate to the semiconductor's surface and react with adsorbed molecules to generate such reactive species as H_2_O_2_, superoxide anion radicals (·O_2_^−^) and hydroxyl radicals (·OH).^2,3^ These species are very strongly oxidative and highly reactive agents, which can degrade in a non-selective way pollutants until their complete mineralization. The principal function of this process is to produce as many radicals as possible for a given energy received by the semiconductor. However, some problems remain. They concern low kinetics and low capacity to absorb photons particularly in the UV range, producing a low photocatalytic efficiency for pollutant degradation.

The main problem of the semiconductors studied in the literature for this process is the photo-sensibility to the ultraviolet or near visible irradiation. The UV region that initiates the photocatalytic process accounts for only 5% of the total available solar flux reaching the surface of the earth.^4^ This represents a maximal irradiation available for the solar catalytic process of 50 W_UV_ m^−2^. Moreover, the overall photocatalytic efficiency obtained with the semiconductors most widely used as catalysts in the literature are relatively weak, of the order of a few percent.^5,6^ In this context, it is very important to use efficiently the sunlight in its totality. By contrast, photocatalytic capacities under UV irradiation are higher than those under solar irradiation: a factor of 10 is observed between these two irradiations spectral range. The principal catalysts used in the literature are effective in the UV range but their yield is very low for solar applications.^7–9^ Therefore, the objective is either to enhance photocatalytic efficiency in the UV range by restricting the recombination process, or to increase photons usable for producing radicals by employing catalysts photosensitive to the visible light. The electron–hole recombination pairs and low photocatalytic efficiency in the photocatalytic reactions hinder the process of photocatalytic degradation. Different methods have been studied in the literature to help decrease the electron–hole recombination rate and/or increase the photocatalytic efficiency of photocatalysts.^2,3,5,10^ The development of innovative nanocatalysts, which are effective in the exploitation of solar radiation is therefore a major challenge.

At present, various metal oxide catalysts, such as WO_3_, ZnS, SnO_2_ and CdS^11–14^ are limited in their application by various constraints, *e.g.* toxicity, chemical instability and/or electron–hole recombination. The two main catalysts that have been widely reported in the literature are ZnO and TiO_2_.^15–17^ ZnO is a promising catalyst for the photocatalytic process due to its photosensitivity, its strong oxidizing capacity, its non-toxicity, its large band gap of 3.37 eV and its excellent chemical and mechanical stability.^18,19^ However, ZnO has several weaknesses, such as the fast recombination rate of the photo-generated electrons and holes and a low efficiency in photocatalytic reactions, which obstruct the photocatalytic degradation mechanism.^2,20^ To address these limitations, modifications of the ZnO structure by doping and/or alloying with different elements have been proposed to improve the photocatalytic activity. ZnO is doped and/or alloyed with many elements, such as Ce,^8^ Mg,^9^ Al,^21^ I,^22^ and Cu.^23^ In recent years, the doping and/or alloying of ZnO with transition metals,^24,25^ alkaline-earth metals^26^ and alkaline metals^25,27^ showed that it is possible to increase the photocatalytic activity under visible light and full solar light irradiation, introducing additional energy levels in the band gap of ZnO and thus modifying its electrical and optical properties.^28,29^ It has also been demonstrated that the doping enhances the charge separation between the photo-generated electrons and holes, decreases the recombination of electron–hole pairs and lengthens the lifetime of electrons and holes.^19,30^ These parameters are directly responsible for photocatalytic efficiency. Among the different alloys commonly studied in the literature, little attention has been devoted to studying the effect of calcium as a dopant or alloying element of ZnO on its photocatalytic activity under solar irradiation.

The present study is focused on the synthesis and characterization of Ca-alloyed ZnO elaborated using different calcium precursors (CaSO_4_, CaCl_2_, Ca(NO_3_)_2_ and CaCO_3_) at different molar ratios to enhance the photocatalytic efficiency. All these catalysts are synthesized by a single sol–gel process.^31^ The structural and morphological properties of samples have been investigated by means of Scanning Electron Microscopy (SEM), Transmission Electron Microscopy (TEM), Energy Dispersive X-ray Spectrum (EDS), X-ray Diffraction (XRD), Raman Spectroscopy and Electron Paramagnetic Resonance (EPR). The photocatalytic degradation of pyrimethanil has been studied to evaluate the influence of precursors and the composition of the samples on the photocatalytic activities under solar irradiation. This allows the photocatalytic efficiency under solar irradiation (computed as the number of degraded molecules divided by the effective photons absorbed available for the photocatalytic reaction) to be determined.

## 2. Experimental

The chemical reagents used in this work are zinc acetate dehydrate [Zn(OOCCH_3_)_2_·2H_2_O], ethanol (C_2_H_5_OH), methanol (CH_3_OH), calcium sulfate (CaSO_4_) or calcium chloride (CaCl_2_) or calcium carbonate (CaCO_3_) or calcium nitrate (Ca(NO_3_)_2_). All of these reagents are purchased from Aldrich Chemical Company and are used without further purification.

### 2.1. Synthesis of Ca-alloyed ZnO

Calcium alloyed ZnO with a calcium sulfate precursor is prepared by sol–gel method using supercritical drying. In a typical preparation procedure, 16 g of zinc acetate dehydrate is first dissolved in 112 ml of methanol under continuous stirring for 15 min at room temperature. An adequate quantity of calcium sulfate, corresponding to [Ca]/[Zn] atomic ratios of 0, 0.01, 0.05, 0.10, 0.15 and 0.20 is added. After 5 min under magnetic stirring, the solution is placed in an autoclave of 1 l capacity to realize the drying under supercritical conditions for ethanol (*T*_c_ = 516 K, *P*_c_ = 63.3 bars). Then, the obtained nanoparticles are heated in a furnace in air at 573 K for 2 hours to enhance the powder crystallinity. Nanoparticles of undoped ZnO and Zn_1−*x*_Ca_*x*_O elaborated with the three other precursors are also prepared following the same procedure.

### 2.2. Characterization

The morphology of the Zn_1−*x*_Ca_*x*_O nanoparticles elaborated with different calcium precursors has been firstly observed using SEM (model SEM-FEG, Hitachi S-4500) and TEM (model JEM-100CX) operating at an acceleration voltage of 100 kV. The identification of chemical elements in the nanopowders has been performed by EDS (model EDS KEVEX Si(Li) assisted software Brüker). The structural properties of samples has then been investigated by XRD with an X'pert powders Philips diffractometer, using the Cu-Kα radiation (*λ* = 1.5418 Å). The diffractograms have been collected at 2*θ* in the range of 10° to 70° with 0.02° step. The crystallite size has been determined from XRD data using the Debye–Scherrer's formula. The formation of the ZnO phase has been confirmed by Raman spectroscopy measurements using a Horiba Jobin Yvon-LabRAM ARAMIS spectrophotometer equipped with diode laser (*λ* = 473 nm) and with power fixed at 16 mW. The EPR method has been used to characterize paramagnetic defects. All EPR measurements have been carried out using a Bruker EMX X-band EPR spectrometer equipped with a 9.8 GHz field modulation unit at room temperature. The resonance has been optimized for the amplitude modulation, frequency modulation, gain, microwave power, time-constant and time conversion. The amount of powder used for all measurements was the same. Additionally, a standard field marker (diphenyl-picrylhydrazyl: DPPH polycrystalline with *g* = 2.0036) has been used for the calibration of the resonance magnetic field values and the determination of the exact *g*-factor of the resonance lines. The shape and area of the EPR spectra have been analyzed using standard numerical methods.

### 2.3. Photocatalytic efficiency

**Fig. 1:**
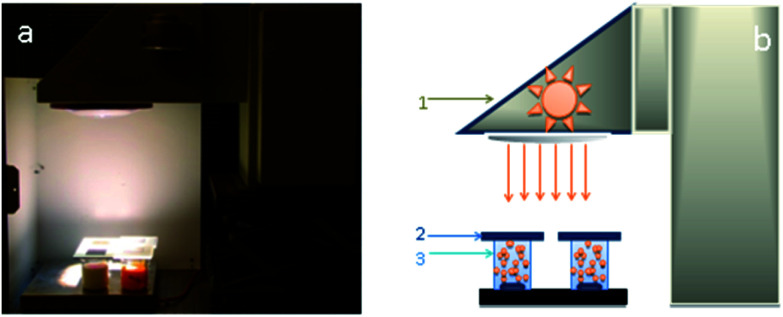
(a) Photograph of the experimental set-up and solar simulator and (b) experimental set-up for the measurement of pyrimethanil degradation kinetic equipped with a source simulating solar irradiation (1), glass optical filters (Edmond Optics filters) (2), four tank reactors (volume of 0.25 l, irradiated surface of 0.0025 m^2^) containing the suspension of catalysts (3).

A measuring bench has been set-up to evaluate the number of molecules degraded by the nanocatalysts under different irradiation conditions. This bench comprised a solar source, optical filters to control the spectral range applied and a reactor containing the catalyst suspensions (Fig. 1). The solar source is made up of a Newport/Oriel solar simulator of 1000 W m^−2^ power and an AM1.5 solar spectrum. To assess to the real number of photons available for photodegradation of the catalysts on different spectral fields, optical filters provided by Edmund Optics are placed between the solar simulator and the beaker with suspensions (Fig. 1b). The three filters used allow to work either in the ultraviolet spectral range from 250 to 400 nm with a power of 46 W m^−2^, or in the visible spectral range from 400 to 560 nm with a power of 315 W m^−2^, or else with a power of 408 W m^−2^ in the solar range from 250 to 560 nm. The reactor is a set of four beakers of 0.25 l volume containing the target molecule to degrade and 0.3 g of nanocatalyst suspensions under magnetic stirring. Defining in a previous work, a optimal catalyst concentration of 0.3 g l^−1^ is chosen for all experiments.^32^ The irradiated surface is 0.0025 m^2^. From this configuration, the photocatalytic efficiency of different nanocatalysts is measured and compared under controlled irradiation conditions. The target molecules selected to evaluate the photocatalytic activity of nanocatalysts is pyrimethanil which is present as a residue in water and food and is toxic to human and aquatic life.^33,34^ Pyrimethanil belongs to the aniline–pyrimidine class of fungicides and has been used worldwide before harvest for foliar application or post-harvest for fruits treatment.^35^ It is used to cure diseases caused by Botrytis, Monilinia, *Venturia pyrina* and other pathogens. Pyrimethanil is not considered readily biodegradable because its half-life is estimated at 8.9–21 days in water. The pyrimethanil and byproducts coming from degradation of the target molecule are tracked by UV-vis spectrophotometer (Thermo scientific evolution 600 UV-vis) using an absorption wavelength of 265 nm. The measurement of degradation kinetics was carried out at an initial concentration of 12 mg l^−1^. Each sample is filtered and measured in a quartz tank with an optical length of 10 mm. Tests are repeated to ensure the results repeatability, hence all the photocatalytic results presented in this work are the mean of three replicates for each samples. Photocatalytic efficiency is used to discuss and explain the obtained results. The efficiency is the variation in the number of photoconverted molecules as the number of accumulated photons received at the photo reactor surface. A previous study^32^ has shown the quantity of adsorbed molecules onto the catalyst is negligible and on other hand, the photolysis can be negligible compared to photocatalytic mechanism.

## 3. Results and discussion

### 3.1. Characterization of the photocatalyst

**Fig. 2:**
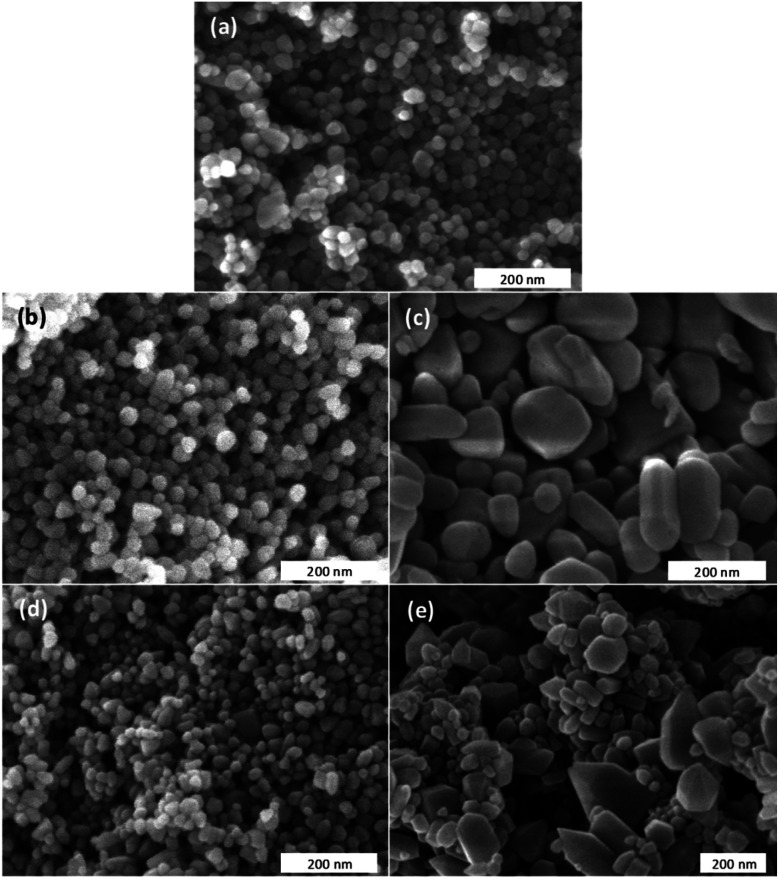
Scanning electron microscopy images of differently elaborated catalysts calcined in air at 573 K (a) undoped ZnO, (b) Zn_0.90_Ca_0.10_O with a CaSO_4_ precursor, (c) Zn_0.90_Ca_0.10_O with a Ca(NO_3_)_2_ precursor, (d) Zn_0.90_Ca_0.10_O with a CaCO_3_ precursor and (e) Zn_0.90_Ca_0.10_O with a CaCl_2_ precursor.

The morphology and the size of the undoped ZnO and of the Zn_1−*x*_Ca_*x*_O nanoparticles have been investigated by SEM. Due to the similarities in terms of size and shape between all calcium contents, authors decided to show only *x* = 10%. Fig. 2 show SEM images of undoped ZnO and Ca-alloyed ZnO catalysts elaborated with the four precursors (CaSO_4_, CaCl_2_, Ca(NO_3_)_2_ and CaCO_3_) for a calcium contents of *x* = 0.10 and calcined in air at 573 K. Fig. 2a, b and d show that undoped ZnO and Zn_0.90_Ca_0.10_O nanoparticles elaborated with a CaSO_4_ and a CaCO_3_ precursors respectively, exhibit mainly quasi-spherical crystallites shapes, with a particle size distribution relatively low and homogeneous centered at 30 nm. The size of these samples is estimated in the range of 20–40 nm. It should be noted that the crystallites size obtained with a CaCO_3_ precursor is lower than the one of ZnO nanoparticles. Also, it can be seen in Fig. 2c and e that the Zn_0.90_Ca_0.10_O nanoparticles prepared with a Ca(NO_3_)_2_ and CaCl_2_ precursor respectively have hexagonal or spherical shapes. These two SEM images show also the presence of hexagonal sticks shape. These particles have a size, which varies from 20 to 200 nm. It is important to note that the shape and crystallite size of the particles depend on the nature of precursors used during elaboration.

**Fig. 3:**
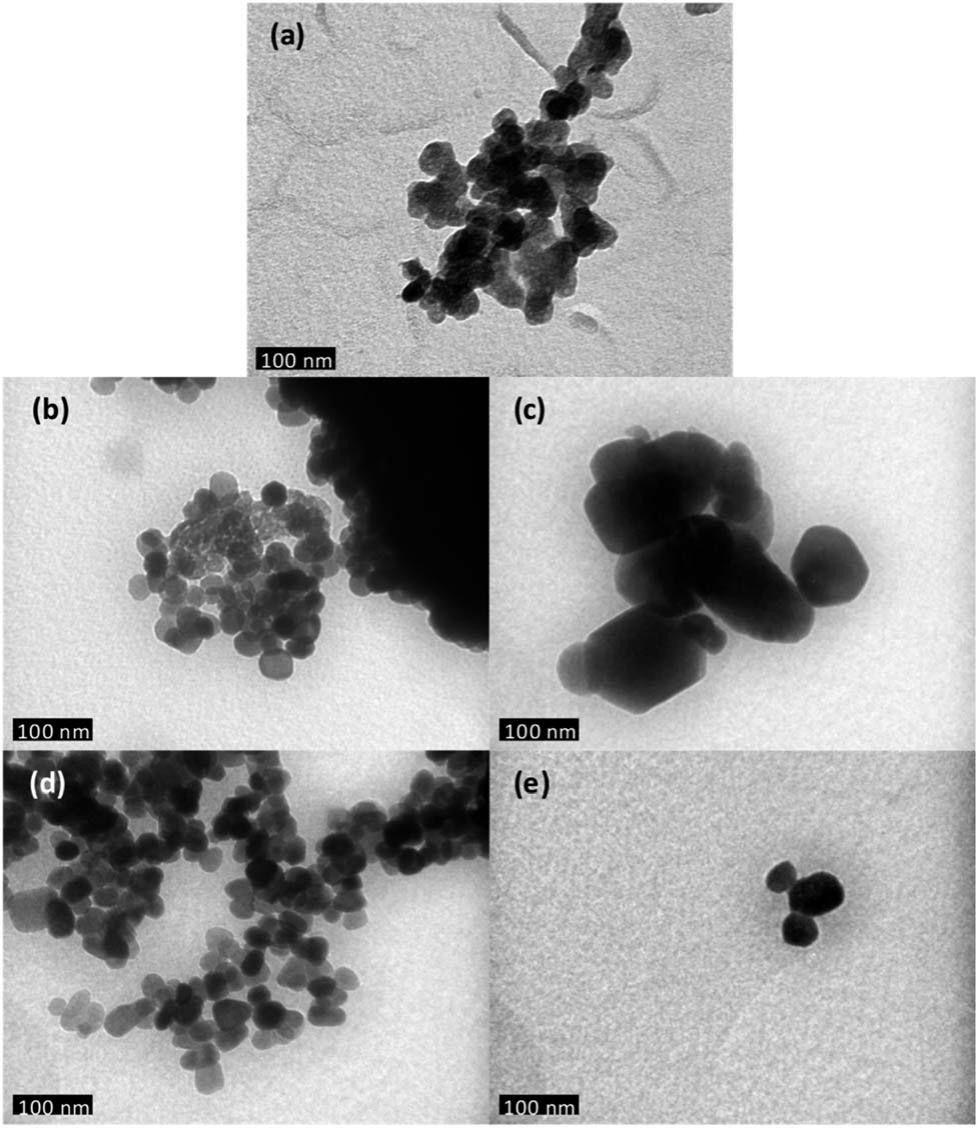
Transmission electron microscopy images of different catalysts elaborated by sol–gel method and calcined in air at 573 K (a) undoped ZnO (b) Zn_0.90_Ca_0.10_O with a CaSO_4_ precursor, (c) Zn_0.90_Ca_0.10_O with a Ca(NO_3_)_2_ precursor, (d) Zn_0.90_Ca_0.10_O with a CaCO_3_ precursor and (e) Zn_0.90_Ca_0.10_O with a CaCl_2_ precursor.

TEM has been performed in order to further carry out the analysis of the crystallites size and morphology. The observation of undoped ZnO and Zn_1−*x*_Ca_*x*_O nanoparticles elaborated with a CaSO_4_, a CaCl_2_, a Ca(NO_3_)_2_ and a CaCO_3_ precursor with *x* at 0.10 is presented in Fig. 3. The undoped ZnO and the Zn_0.90_Ca_0.10_O nanoparticles with a CaSO_4_ and a CaCO_3_ precursor, shown respectively in Fig. 3a, b and d, have a quasi-spherical morphology with an average size between 20 and 40 nm. In contrast, for other precursors in Fig. 3c and e, a quasi-spherical, stick or even hexagonal shapes are present. The average nanoparticles size varies from 30 to 200 nm. These results again confirm that the nature of the precursor used during the synthesis have an influence on nanoparticles size and shape in agreement with those obtained by SEM.

**Fig. 4:**
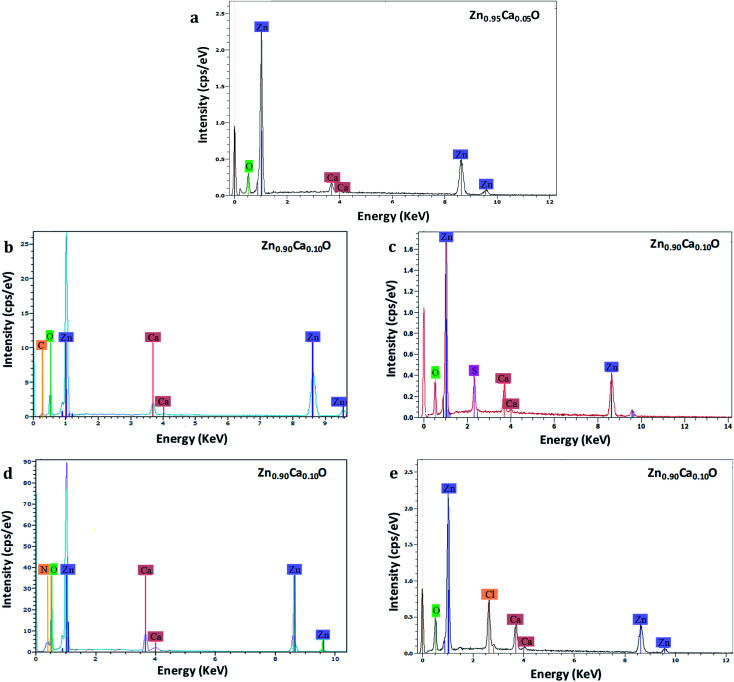
EDS spectra of Zn_0.95_Ca_0.05_O nanoparticles elaborated with CaCO_3_ precursor (a) and Zn_0.90_Ca_0.10_O nanoparticles elaborated from CaCO_3_ (b), CaSO_4_ (c), Ca(NO_3_)_2_ (d) and CaCl_2_ (e) precursors. The nanoparticles are calcined in air at 573 K.

EDS analysis has been performed to measure the chemical compositions of Zn_1−*x*_Ca_*x*_O nanoparticles elaborated with different precursors with *x* varying from 0 to 0.20. This analysis was replicated three times at three different locations on each samples. Fig. 4 shows the EDS spectra of the Zn_1−*x*_Ca_*x*_O nanoparticles. EDS confirms the presence of zinc, calcium, oxygen and another element. The peaks observed at 0.5, 1.0 and 3.7 keV are due to the presence of O, Zn and Ca respectively.

However, for the Zn_0.90_Ca_0.10_O nanoparticles elaborated with CaSO_4_, CaCl_2_, Ca(NO_3_)_2_ and CaCO_3_ precursors, supplementary elements identified as sulphur, chlorine, nitrogen and carbon are attributed to their respective precursors. Furthermore, for the Zn_0.95_Ca_0.05_O nanoparticles elaborated with the CaSO_4_ precursor, no carbon from the precursor is found. The most relevant results are summarized in Table 1 for calcium contents of 5% and 10%. The elemental composition values for Zn_1−*x*_Ca_*x*_O nanoparticles at *x* = 0.10 elaborated with a CaSO_4_ precursor show that stoichiometry is not respected. In contrast, the quantity of calcium present in the samples is in agreement with the initial content of 10% at ±1%, representing the instrumental error. However, the atomic percentage of sulfur and calcium are the same. In this case, the presence of sulfur indicates probably that calcium is not fully inserted in the ZnO matrix. It can be seen from Table 1 that the Zn_1−*x*_Ca_*x*_O sample with *x* = 0.10 elaborated with a CaCl_2_ precursor is not stoichiometric. A significant presence of chlorine and calcium is observed, as well as a high deficiency in zinc. The elemental compositions values obtained for the Zn_0.90_Ca_0.10_O nanoparticles elaborated with a Ca(NO_3_)_2_ precursor also show a non-respect of the stoichiometry. This is due to the presence of the nitrogen element in the elaborated catalyst. In contrast, the elemental chemical compositions obtained for the Zn_1−*x*_Ca_*x*_O nanoparticles with *x* = 0.05 and *x* = 0.10 elaborated with a CaCO_3_ precursor are quasi-stoichiometric. The atomic percentage of calcium is in agreement with the elaboration conditions of 5% and 10% at ±1%. Moreover, a low presence of carbon is observed for *x* = 0.10. This confirms the presence of calcium in the ZnO matrix. It is important to note that the precursor plays a role on the stoichiometry and on the insertion in the ZnO matrix.

**Table 1:** EDS results of Zn_1−*x*_Ca_*x*_O nanoparticles elaborated with the four precursors at different calcium content

Catalyst	Precursor	[Ca]/[Zn]	Zinc (at%)	Oxygen (at%)	Calcium and other elements (at%)
Zn_0.95_Ca_0.05_O	CaCO_3_	0.05	47.02	50.37	Ca: 2.61
Zn_0.90_Ca_0.10_O	CaCO_3_	0.10	44.27	50.25	Ca: 4.44 C: 1.04
	CaSO_4_	0.10	44.08	44.47	Ca: 5.55 S: 5.90
	CaCl_2_	0.10	26.65	51.56	Ca: 8.34 Cl: 13.45
	Ca(NO_3_)_2_	0.10	50.06	40.08	Ca: 5.10 N: 4.76

**Fig. 5:**
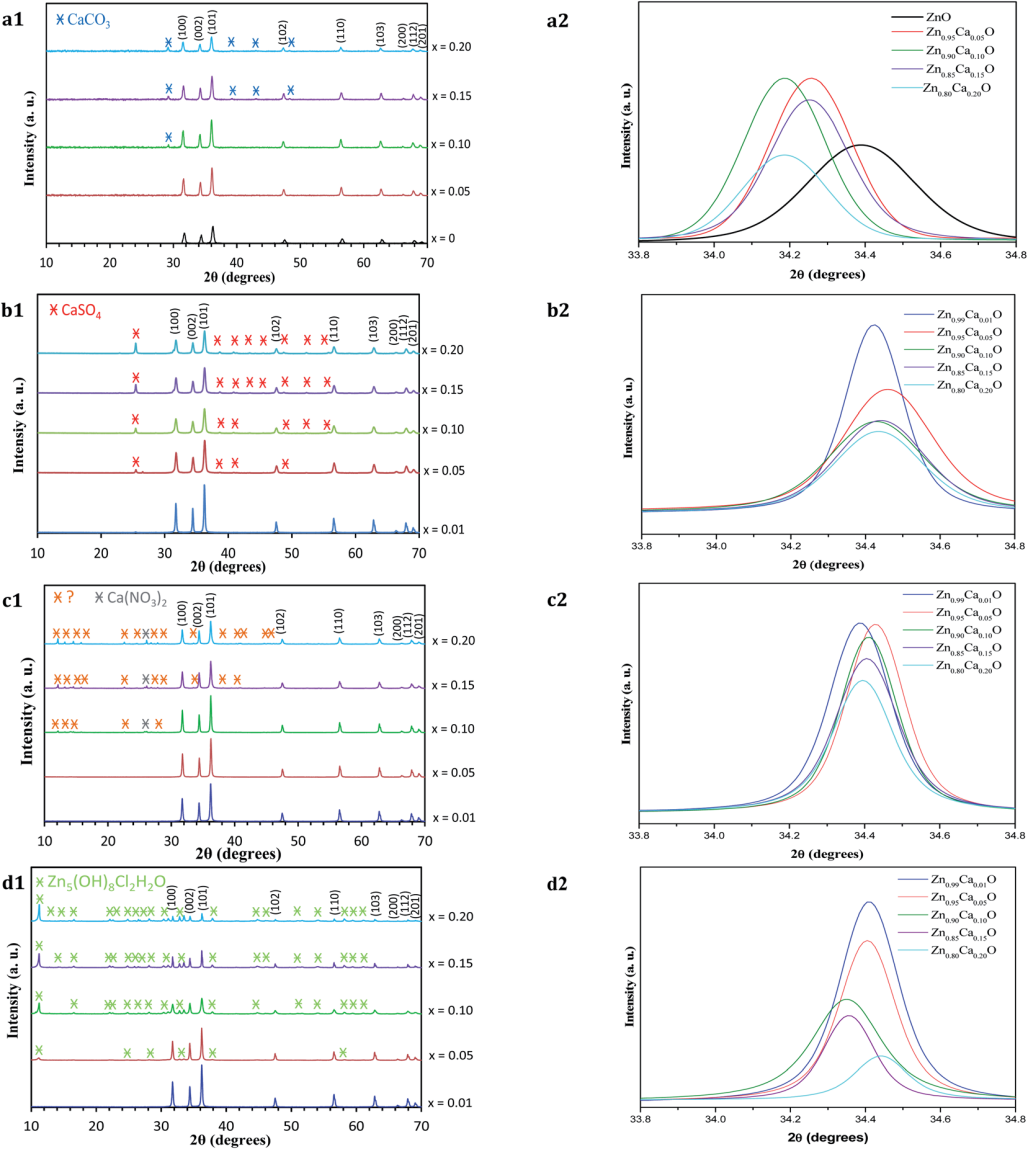
(1) XRD patterns and (2) their corresponding zoom in on the (002) diffraction peak of Zn_1−*x*_Ca_*x*_O nanoparticles elaborated with the four different precursors at different calcium content (*x* = 0 and 0.01 ≤ *x* ≤ 0.20) and calcined in air at 573 K: (a) CaCO_3_, (b) CaSO_4_, (c) Ca(NO_3_)_2_ and (d) CaCl_2_ precursors. The XRD pattern of undoped ZnO nanoparticles is also shown.

The structure of the undoped ZnO and Zn_1−*x*_Ca_*x*_O samples elaborated with different precursors (CaSO_4_, CaCl_2_, Ca(NO_3_)_2_ and CaCO_3_) at different contents has been investigated using X-ray powder diffraction. The XRD patterns of Zn_1−*x*_Ca_*x*_O nanoparticles for a number of *x* values between 0 and 0.20 are shown in Fig. 5.

For all the samples, the positions and relative intensities of the diffraction peaks are in good agreement with powder diffraction standards data (JCPDS no. 36-1451), corresponding to the hexagonal wurtzite structure of ZnO (space group *P*6_3_*mc*) with lattice constants of *a* = *b* = 3.25 Å, *c* = 5.207 Å. The main peaks are identified as (100), (002), (101), (102), (110), (103), (200), (112) and (201) reflexion planes of ZnO, respectively. For our samples, the (002) diffraction line is narrower than the (101) and (100) lines. This indicates that the crystalline shape is asymmetrical. When the calcium content increases in the ZnO matrix, the degree of crystallinity of the nanoparticles decreases. Indeed, at *x* ≥ 0.10 in the case of the CaCO_3_ and Ca(NO_3_)_2_ precursors and *x* ≥ 0.05 with the CaSO_4_ and CaCl_2_ precursors, other peaks which do not belong to the wurtzite structure begin to appear. The relative intensity and number of these peaks increases with calcium content. On Fig. 5a1, c1 and b1, the appearance of the secondary phases for Zn_1−*x*_Ca_*x*_O when *x* ≥ 0.10 with the CaCO_3_ and Ca(NO_3_)_2_ precursors and *x* ≥ 0.05 with the CaSO_4_ precursor can be attributed to the presence of the precursor itself.^36–38^ This is probably due to the limited solubility of the calcium atoms substituting the Zn interstitial sites of the ZnO matrix. In contrast, the secondary phases observed in Fig. 5d1 are identified using the JCPSD 07-0155 database^39,40^ and attributed to zinc hydroxyl compound Zn_5_(OH)_8_Cl_2_–H_2_O. It is formed from a chemical reaction between the zinc and calcium (CaCl_2_) precursor during the catalyst synthesis. However, the phases presented in Fig. 5c1 are not referenced in the literature.

Furthermore, the lattice parameters *a* = *b* and *c* determined from the positions of the (001) and (002) diffraction peaks for the Zn_1−*x*_Ca_*x*_O (0.01 ≤ *x* ≤ 0.20) elaborated with the CaSO_4_, CaCl_2_ and Ca(NO_3_)_2_ precursors are relatively close to those of ZnO. Only with a CaCO_3_ precursor, an increase of lattice parameters is observed. This is due to the insertion of calcium in the ZnO matrix. In fact, since the atomic radius size of the calcium ion (1.94 Å) is larger than that of the zinc ion (1.42 Å), dislocations appeared and led to a local distortion of the lattice parameter.^41^ No modification of the ZnO wurtzite structure is generated.

Nevertheless, the enlargement of (002) diffraction peak is showed in Fig. 5a2 for Zn_1−*x*_Ca_*x*_O (*x* = 0 and 0.05 ≤ *x* ≤ 0.20). Only in the case of CaCO_3_, a shift toward a lower 2*θ* degrees is observed when the Ca content increases. This shift indicates that the Ca^2+^ ions substituted the zinc sites along the *c*-axis without modification of the ZnO wurtzite lattice.^42–44^ In contrast, on Fig. 5b2–d2 which represent respectively Zn_1−*x*_Ca_*x*_O (0.01 ≤ *x* ≤ 0.20) elaborated with the CaSO_4_, Ca(NO_3_)_2_ and CaCl_2_ precursors, the shift of the (002) diffraction peak is relatively low. This indicates that calcium isn't fully integrated into the ZnO matrix. All these results are in agreement with the ones obtained by EDS and presented above. It is important to note that the precursor plays a role on the insertion in the ZnO matrix.

The average crystallite size *G* of the different samples has been calculated using Debye–Scherrer's formula given in eqn (1).^45^1
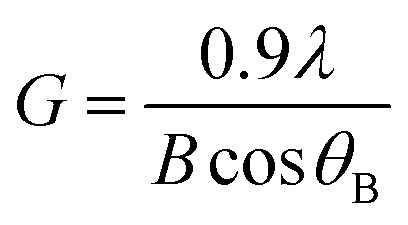
where *λ* is the X-ray wavelength (1.5418 Å), *B* is the line width at half maximum (FWHM) of the XRD peak and *θ*_B_ is the Bragg diffraction angle. The average crystallite size obtained for Zn_1−*x*_Ca_*x*_O (0.05 ≤ *x* ≤ 0.20) samples elaborated with the CaSO_4_ and CaCO_3_ precursors varies from 22 to 34 nm and is 25 nm for the undoped ZnO. In these two cases, it is interesting to note that the presence of precursors does not influence the crystallites size. In contrast, the average crystallite size for Zn_1−*x*_Ca_*x*_O (0.01 ≤ *x* ≤ 0.20) elaborated with the two other precursors varies from 39 to 56 nm. The crystallite size is much greater compared to that of undoped ZnO. Therefore, the nanoparticles size variation is directly influenced by the precursor chosen during their synthesis. These results are in good agreements with the crystallites size determined by SEM and TEM.

**Fig. 6:**
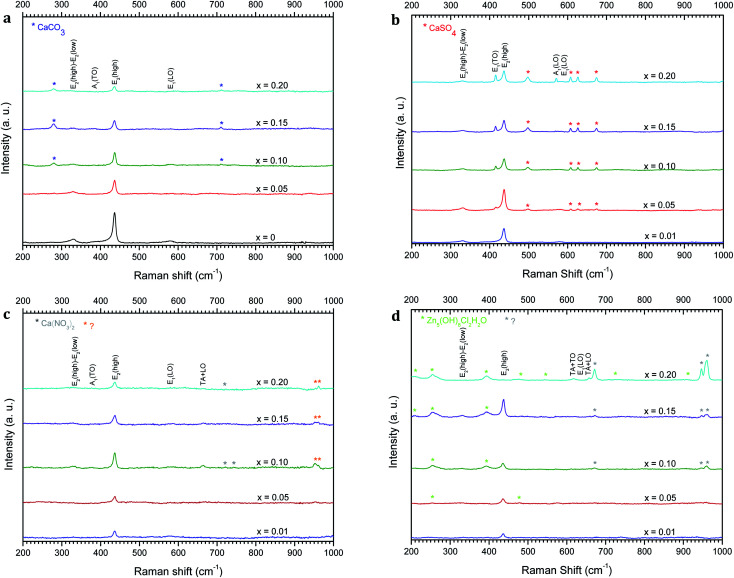
Raman spectra of Zn_1−*x*_Ca_*x*_O (*x* = 0 and 0.01 ≤ *x* ≤ 0.20) nanoparticles elaborated from CaCO_3_ (a), CaSO_4_ (b), Ca(NO_3_)_2_ (c) and CaCl_2_ (d) precursors. The nanoparticles are calcined in air at 573 K.

Raman analysis has been performed to further obtain more information on the structural compositions of the undoped ZnO and Zn_1−*x*_Ca_*x*_O nanoparticles elaborated with different precursors (CaSO_4_, CaCO_3_, Ca(NO_3_)_2_ and CaCl_2_) at different calcium contents ranging from *x* = 0 to *x* = 0.20. Raman spectra have been acquired at room temperature and are represented as a function of the wavenumber in the range of 200–1000 cm^−1^ in Fig. 6. The several peaks shown at 331, 382, 415, 437, 574 and 584 cm^−1^ are attributed to the optical phonon of ZnO.^46–48^ While the 331 cm^−1^ and 382 cm^−1^ peaks correspond to the second order Raman (multiple phonon processes) E_2_ (high)-E_2_ (low) and A_1_ (TO) modes respectively. The intense peak at around 437 cm^−1^ is assigned to the high frequency branch of the E_2_ (high) vibrational mode. This mode corresponds to oxygen motion and is a characteristic of the hexagonal wurtzite phase of ZnO.^49^

Modes are identified for a calcium content of *x* = 0.10, except for the Zn_1−*x*_Ca_*x*_O elaborated with the CaSO_4_ and CaCl_2_ precursors where the content is *x* = 0.05. The Zn_1−*x*_Ca_*x*_O elaborated with the CaSO_4_, CaCO_3_ and Ca(NO_3_)_2_ precursors, presented supplementary vibrational modes attributed to the calcium precursor used during the sol–gel synthesis.^37,52,53^

Moreover, the crystallinity of the nanoparticles is directly correlated to the intensity and width of E_2_(high) mode.^50^ In Fig. 6a, Fig. 6c and Fig. 6b, Ca-alloyed ZnO with a calcium content *x* > 0.10 for the CaCO_3_ and Ca(NO_3_)_2_ precursors and *x* > 0.05 for the CaSO_4_ precursor respectively, an intensity decrease of the E_2_(high) peak is observed. In the case of the CaCl_2_ precursor (Fig. 6d), this appear for a calcium content *x* > 0.15. This indicates a degradation of the material's crystallinity. The peak at 584 cm^−1^ attributed at E_1_(LO) appears for *x* ≥ 0.10. This vibrational mode implies the presence of structural defects assigned to the oxygen vacancies, Zn interstitials and also impurities.^51^ Furthermore, the supplementary vibrational the sol–gel synthesis.^37,52,53^ Furthermore, the vibrational modes presented in Fig. 6c at 951 and 961 cm^−1^ for this precursor are not referenced in the literature. On the other hand, the supplementary vibrational modes for the CaCl_2_ precursor are attributed to the zinc hydroxyl compound Zn_5_(OH)_8_Cl_2_–H_2_O.^54–56^ These results are in a perfect agreement with the one obtained by XRD.

### 3.2. Photocatalytic efficiency

Most studies in the literature are performed under UV irradiation, which is not representative of real solar irradiation.^57,58^ Thus, in order to achieve representativeness of the real conditions, the set-up including UV and visible irradiation described above in paragraph 2.3 has used. The objective of this study is to define the photocatalytic performances of nanocatalysts under solar irradiation and to compare them in terms of their photocatalytic efficiency. In the heterogeneous photocatalysis processus, dissociating the “event” and defining the number of incident photons responsible for the photocatalytic process are complex matters.^5^ According to the quantum yield introduced by Serpone for homogeneous photocatalysis,^5^ photocatalytic efficiency (eqn (2)) is defined simply by the number of given events (mineralization, molecules photoconverted or radicals production) relative to the total number of photons incident on the reactor wall. In this work, the photocatalytic efficiency *Φ* indicates the number of molecules degraded divided by the number of incident photons absorbed by the reactant(s) or by the photocatalysts for all of the irradiation. Thus,2
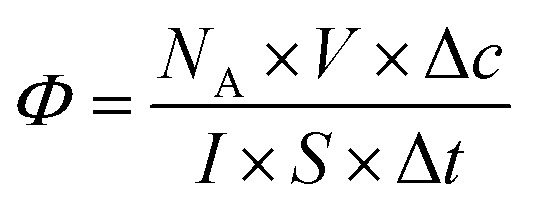
where *N*_A_ is the Avogadro constant (mol^−1^), *V* the volume of the photoreactor (l), Δ*c* the difference between the initial and the final concentration (mol l^−1^), *I* the flux density measured at the surface of the reactor (number of photons m^−2^ s^−1^), Δ*t* the irradiation time (s) and *S* the receptor surface (m^2^). The measured photons on the reactor represent the efficient photons received under solar irradiation in the spectral range from 250 to 1000 nm.^35^ The photocatalytic efficiencies measurements were replicated a minimum of three times on each sample.

#### 3.2.1. Effect of the precursor content

**Fig. 7:**
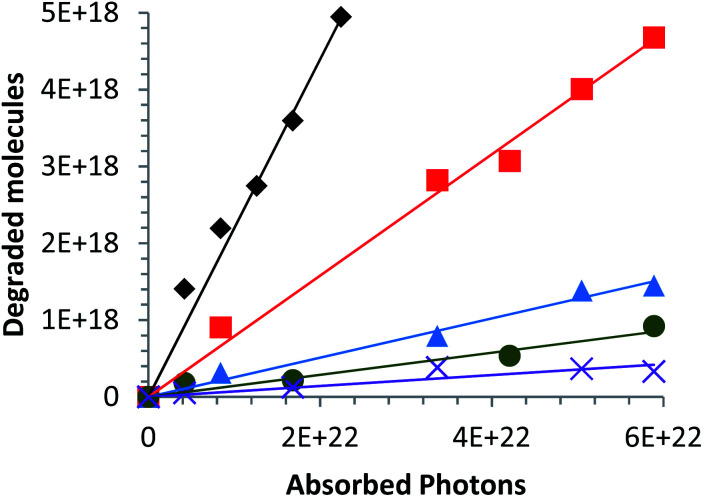
Variation in the number of degraded molecules as a function of the number of absorbed photons in the ultraviolet and visible spectral range (250–700 nm) for different catalysts elaborated with a CaCl_2_ in suspension: (◆) Zn_0.99_Ca_0.01_O, (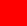) Zn_0.95_Ca_0.05_O, (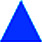) Zn_0.90_Ca_0.10_O, (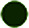) Zn_0.85_Ca_0.15_O and (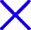) Zn_0.80_Ca_0.20_O. The Ca-alloyed ZnO samples are calcined in air at 573 K.

A first study has been performed on the influence of the calcium content on the photocatalytic efficiencies of Zn_1−*x*_Ca_*x*_O nanoparticles elaborated with a CaCl_2_ precursor at different compositions ranging from *x* = 0.01 to *x* = 0.20. Fig. 7 represents the variation of photoconverted molecules as a function of the number of photons received in the spectral range from 250 to 700 nm. The slopes of these lines correspond to the photocatalytic efficiency of each catalyst. Fig. 7 shows that the nanoparticles are photosensitive under solar irradiation. In that case, the photocatalytic efficiency decreases with increasing calcium content.

This is due to the increase of the secondary phases present in the material and identified in part 3.1 (Fig. 6d). According to Linder *et al.*, inorganic ions such as Cl^−^ can be harmful to the photocatalyst efficiency.^59^ The optimal alloy percentage for the CaCl_2_ precursor is 1%. The performance of these nanoparticles is now compared to those of the nanoparticles elaborated with the other precursors.

#### 3.2.2. Effect of the precursor nature

**Fig. 8:**
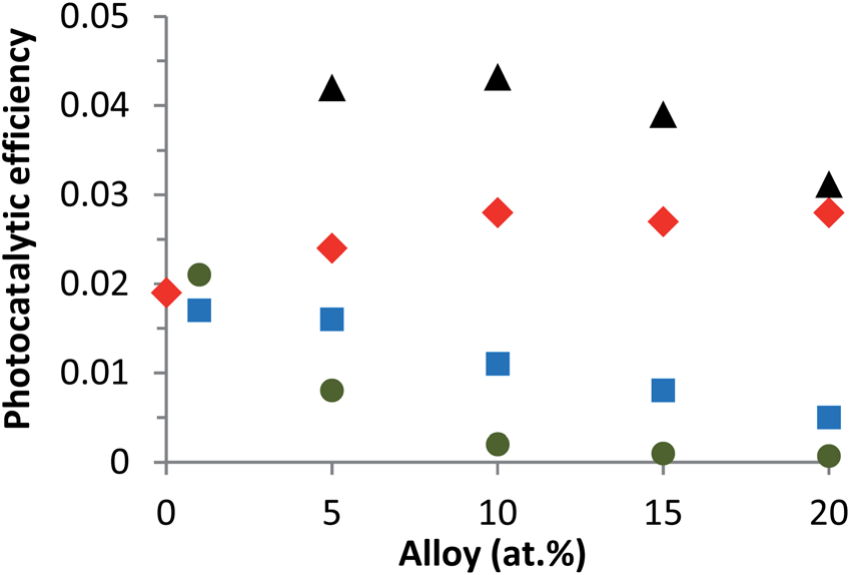
Variation of the photocatalytic efficiency as a function of the alloy content from the UV and visible spectral range (250–700 nm) for different catalysts calcined in air at 573 K in suspension: (▲) Zn_1−*x*_Ca_*x*_O with a CaCO_3_ precursor, (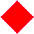) Zn_1−*x*_Ca_*x*_O with a CaSO_4_ precursor, (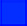) Zn_1−*x*_Ca_*x*_O with a Ca(NO_3_)_2_ precursor and (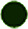) Zn_1−*x*_Ca_*x*_O with a CaCl_2_ precursor.

In second part, the variation of the photocatalytic efficiency under solar irradiation of Zn_1−*x*_Ca_*x*_O nanoparticles elaborated with different precursors and with *x* varying from 0 to 0.20 is shown in the Fig. 8. For all precursors, the photocatalytic efficiency was directly deduced as from eqn (2). As it can be seen from this graph, the photocatalytic results vary drastically depending upon the precursor used. Zn_1−*x*_Ca_*x*_O nanoparticles elaborated with a CaCO_3_ and a CaSO_4_ precursors present similar trends. On one hand, their photocatalytic efficiencies increase with increasing calcium content, reaching a maximum at *x* = 0.10 and exhibiting a plateau for the CaSO_4_ precursor after that, whereas for the CaCO_3_ precursor the efficiency decreases when the calcium content is further increased.

On the other hand, for the two other precursors an increase of calcium content tends to decrease the photocatalytic efficiency. In this case, the decrease is more rapid with a CaCl_2_ precursor. Compared to undoped ZnO, the Ca-alloyed ZnO elaborated with a CaCO_3_ precursor enhances the photocatalytic performances. It's important to note that the photocatalytic efficiency is multiplied by a factor of 2. In the case of the CaSO_4_ precursor, the photocatalytic efficiency stays close to that of undoped ZnO. In contrast, the nanoparticles elaborated using the two others precursors, CaCl_2_ and Ca(NO_3_)_2_, exhibit similar or even worse photocatalytic results than undoped ZnO.

Two key parameters have thus been identified as responsible for the evolution of the photocatalytic performances. The first is the choice of the precursor and the second is the Ca content used for a given precursor. The enhancement of the photocatalytic efficiency for the Zn_1−*x*_Ca_*x*_O nanoparticles elaborated with a CaCO_3_ precursor at *x* ranging from 0.05 to 0.20 is due to the Ca^2+^ ions substituting the Zn interstitial sites of the ZnO matrix. The possible explanation could be that during ZnO doping, enhancements of the photocatalytic activity, by an increase of the dopant content, present as a band gap decrease, leads to an effective lower energy photons absorption. As shown in our previous study, the Ca-doped ZnO presents a lower band gap than the pristine ZnO that is to say, an increase of the absorption to the lower wavelength.^60^ Moreover, the doping created trapping sites which affects the charges carrier lifetime. In this sense, charge recombinations are reduced and lead to a best photocatalytic efficiency.^2,3,61,62^ On the contrary, when higher dopant content is reached, a multiple trapping of charges carriers appears. This leads to an increase of the possibly of the electron–hole recombination and decrease charge carriers which reach the surface to initiate the degradation of molecule, hence decrease of the photocatalytic efficiency.^63,64^ The Zn_1−*x*_Ca_*x*_O catalyst at *x* = 0.10 elaborated with a CaCO_3_ precursor provides the best photocatalytic efficiency. For the Zn_1−*x*_Ca_*x*_O nanoparticles elaborated with a CaSO_4_ precursor, the photocatalytic efficiency is close to that of undoped ZnO because the calcium isn't integrated in the ZnO matrix as shown in Table 1, Fig. 5b2 and 6b. In contrast, the decrease of the photocatalytic efficiency for the two others precursors is due to the presence of secondary phases in the material identified in Fig. 5c1 and d1, Fig. 6c and d. These phases not only belong to the precursor but they are due to the creation of zinc hydroxyl compound. Linder *et al.* have shown that the inorganic Cl^−^ and NO_3_^−^ ions degrade the photocatalytic performances.^59^ These results are in agreement with those from EDS, XRD and Raman spectroscopy presented in part 3.1.

#### 3.2.3. Correlation between intrinsic defects and photocatalytic efficiency

In order to understand the difference in term of photocatalytic efficiency for these different precursors a correlation between the intrinsic default of the nanoparticles and the photocatalytic efficiency has been done. To take the analysis further, the X-band EPR method at room temperature has been chosen to study and quantify the intrinsic defects in the undoped ZnO and Zn_1−*x*_Ca_*x*_O with *x* = 0.10 elaborated with the three precursors (CaSO_4_, CaCO_3_ and CaCl_2_). On the EPR spectra of Zn_0.90_Ca_0.10_O elaborated with the cited precursors, it appears that the nanoparticles present two groups of signals that can be assigned to different defects centers. The first is around *g* ∼ 1.96 and the second is around *g* ∼ 2.00. However, these two signals appear simultaneously with a CaCl_2_ precursor and only one signal is there for the CaSO_4_ and the CaCO_3_ precursors. The signal at *g* ∼ 1.96 originates from defects in the bulk (core), whereas the signal near *g* ∼ 2.00 arises from surface defects (shell).^65^ The main origin of intrinsic defect centers and their assignment are still controversial in the literature and have been so for decades. However, recently core–shell defects have been identified such as negatively charged zinc vacancies (V_Zn_^−^) acting as shallow acceptor and positively charged oxygen vacancies (V_O_^+^) acting as deep donor, respectively.^66^ Supplementary resonance lines are observed for all the nanoparticles elaborated and the lack of information concerning EPR lines in the literature didn't allow us to identify them. We strongly believe that this line belongs to the precursor used during the synthesis as we demonstrated in part 3.1. On the other hand, for the Zn_0.90_Ca_0.10_O catalyst elaborated with a CaCl_2_ precursor the asymmetry of the sextuplet signal is associated to the chlorine isotopes ^35^Cl and ^37^Cl with *I* = 3/2.^67^ This result corroborates with those obtained by EDS, XRD and Raman spectroscopy. Having identified these defects, we have quantified the number of defects present in our nanoparticles of undoped ZnO and Zn_1−*x*_Ca_*x*_O elaborated with a CaSO_4_, a CaCO_3_ and a CaCl_2_ precursor at *x* = 0.10. To this end it is important to note that the surface of spectra obtained from the derivative of the EPR absorption as a function of the magnetic field is directly related to the spin number *N*_s_ in the sampled material. The spin number is calculated experimentally from the integral of the derivative signal of absorption and from the standard sample whose spin number is known.

**Fig. 9:**
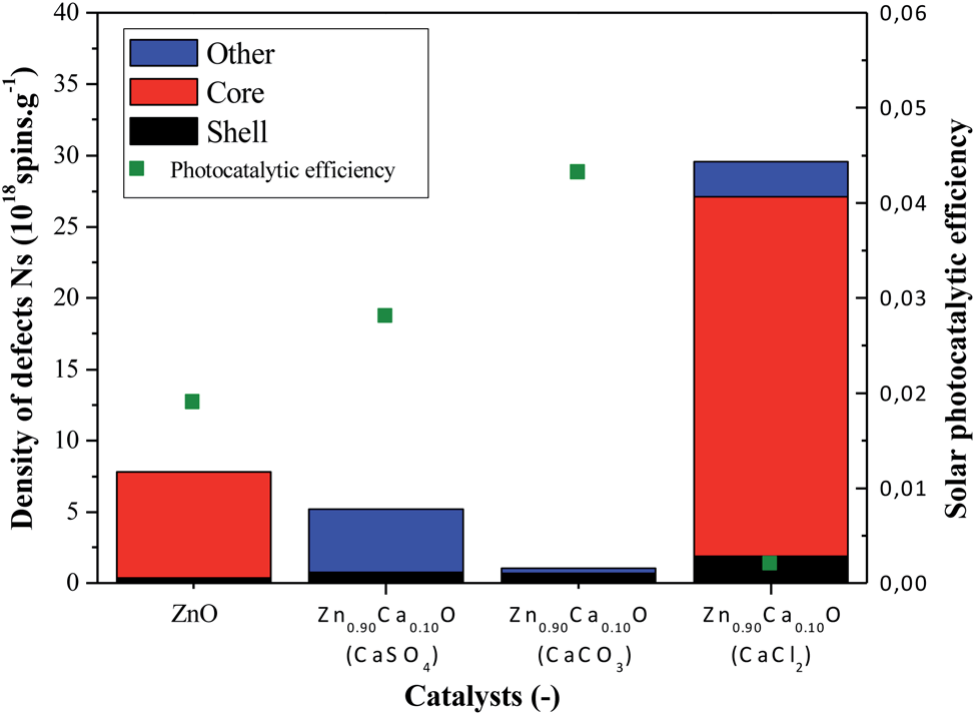
Variation of the density of defects and photocatalytic efficiency under solar irradiation for undoped ZnO and Zn_0.90_Ca_0.10_O nanoparticles with a CaSO_4_, a CaCO_3_ and a CaCl_2_ precursor: (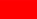) number defects in the core, (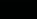) number of defects at the surface (shell), (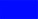) other defects and (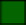) photocatalytic efficiency under solar irradiation.

The number of defects is estimated using the following eqn (3).^68^3
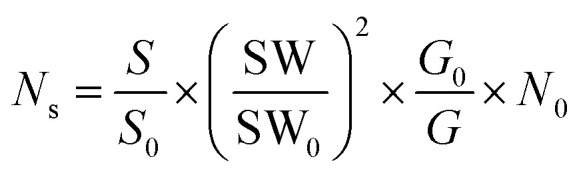
where *N*_s_ is the number of defects, *S* the surface of the integrated signal, SW the sweep width (Gauss) and *G* the gain. The subscript “0” refers to spin number, integral and other conditions used with DPPH. It should be noted that this comparative method requires spectra acquisition under the same conditions of power and amplitude modulation as the standard one. The spin number (*N*_s_) is then reported at its mass (spins g^−1^). The number of defects core, shell and other of undoped ZnO and Zn_0.90_Ca_0.10_O nanoparticles elaborated with a CaSO_4_, a CaCO_3_ and a CaCl_2_ precursor are reported in the Fig. 9. On this same figure is also reported the photocatalytic efficiency under solar irradiation in order to correlate it with the number of defects.

In Fig. 9, a drastic increase of the number of defects can be observed when using the CaCl_2_ precursor. However, the two others precursors caused the number of defects to decrease in comparison with the undoped ZnO. The absence of core defects as well as a low quantity of total defects in the catalysts present the best photocatalytic efficiency. On the other hand, a huge presence of core defects in the Zn_0.90_Ca_0.10_O nanoparticles elaborated with a CaCl_2_ precursor decrease drastically the photocatalytic efficiency.

The best photocatalytic efficiency is obtained for the Zn_0.90_Ca_0.10_O nanoparticles elaborated with a CaCO_3_ precursor. It is important to notice that the core–shell defects in the material play an essential role on the photocatalytic efficiency. The presence of core defects is attributed to negatively charged zinc vacancies representing electron traps, which facilitate charges recombination. The presence of those defects has a harmful effect in photocatalysis. This is also the case for the presence of other defect attributed to Cl^−^ ions in the synthesis using a CaCl_2_ precursor.^59^ The nature of the precursor clearly influences the type and the number of defects on the synthesized catalysts. The synthesis of catalysts with a controlled density of defects is essential to enhance catalysts performances. Therefore, a future study will be done by annealing nanoparticles in a controlled atmosphere such as O_2_, H_2_ or N_2_.

## 4. Conclusions

Zn_1−*x*_Ca_*x*_O nanoparticles elaborated with four calcium precursors (CaSO_4_, CaCO_3_, Ca(NO_3_)_2_ and CaCl_2_) with *x* ranging from 0 to 0.20 have been synthesized by the sol–gel process followed by supercritical drying and have been used as catalyst in photodegradation mechanism of pyrimethanil. The photocatalytic efficiency has been determined, in order to discriminate between these catalysts.

The aim was to study the influence of the calcium precursor on the photocatalytic efficiencies under solar irradiation, taking into account the overall available photons. The photocatalytic results have shown that the Zn_1−*x*_Ca_*x*_O nanoparticles elaborated with a CaCO_3_ precursor at *x* ranging from 0 to 0.20 enhance the photocatalytic efficiency under solar irradiation, whereas in the case of the CaSO_4_ precursor the photocatalytic efficiency is similar to that of ZnO. On the other hand, for the nanoparticles elaborated with the two other precursors the photocatalytic efficiency is degraded. The experimental results obtained in this study lead to the conclusion that the precursor and the calcium content play an essential role on the photocatalytic efficiency.

Characterization of the alloys using a number of complementary techniques shows that only in the case of Zn_1−*x*_Ca_*x*_O nanoparticles elaborated with the CaCO_3_ precursor, calcium ions substituted the zinc interstitial sites in the ZnO matrix. In contrast, for the three other precursors, the presence of secondary phases in the catalyst and the similarly of the lattice parameters in comparison to ZnO indicates that the calcium isn't introduced in the ZnO matrix. Moreover, the number of intrinsic defects in the material decreased when the CaCO_3_ precursor was used. It is shown that a substantial density of total defects (core, shell and other defects) induces a drastic decrease of the catalysts photocatalytic efficiency. In this work, it has been demonstrated that the presence of core defects in the material is harmful in photocatalysis due to electrons–holes charges recombination.

The maximal photocatalytic efficiency has been obtained for Zn_1−*x*_Ca_*x*_O nanoparticles elaborated with a CaCO_3_ precursor at *x* = 0.10, for which we have confirmed the disappearance of core defects and a low presence of shell and other defects. These results are promising for the development of new high-performance photocatalysts.

## Conflicts of interest

There are no conflicts to declare.

## Supplementary Material
